# Eminectomy for Habitual Luxation of the Temporomandibular Joint with Sedation and Local Anesthesia: A Case Series

**DOI:** 10.1155/2016/2505864

**Published:** 2016-10-13

**Authors:** Joe Iwanaga, Yoshiaki Nakamura, Jingo Kusukawa, R. Shane Tubbs

**Affiliations:** ^1^Dental and Oral Medical Center, Kurume University School of Medicine, 67 Asahi-machi, Kurume, Fukuoka 830-0011, Japan; ^2^Seattle Science Foundation, 550 17th Ave, James Tower, Suite 600, Seattle, WA 98122, USA; ^3^Department of Dental and Oral Surgery, Saiseikai Hita Hospital, 643-7 Miwa, Hita, Oita 877-0000, Japan

## Abstract

Eminectomy which is one of the popular and most effective treatments for habitual temporomandibular joint luxation was first described by Myrhaug in 1951. There are few reports which described eminectomy being performed under local anesthesia and conscious sedation. We present a case series of habitual luxation of the TMJ treated by eminectomy performed under local anesthesia and conscious sedation and general anesthesia. Five patients were examined and found to have recurrent luxation of the TMJ. The age of patients ranged from 18 to 93 years. Bilateral eminectomy of the TMJ was performed for two patients, and unilateral eminectomy was performed for three patients. Two were examined under intravenous propofol sedation and local anesthesia, while three patients were examined under general anesthesia. One patient died from ileus one month after surgery. The follow-up period except for the case that died from ileus ranged from 12 to 33 months. No recurrent dislocation of the TMJ has been identified. Based on our experience and two other series in the literature, eminectomy with sedation and local anesthesia can be considered and might be a good option in elderly patients.

## 1. Introduction

Habitual luxation of the temporomandibular joint (TMJ) often occurs in the elderly and often occurs during activities such as yawning and laughing [1]. Treatments for habitual TMJ luxation, which can be conservative or surgical, include eminectomy, which was first described by Myrhaug in 1951 [2]. Although almost all such procedures have been performed under general anesthesia [2, 3] there are a few reports that have described eminectomy being performed under local anesthesia and conscious sedation [4, 5]. We present a case series of habitual luxation of the TMJ treated by eminectomy performed under local anesthesia and conscious sedation and general anesthesia. This study was performed in keeping with the requirements of the Declaration of Helsinki (64th World Medical Assembly, Fortaleza, Brazil, October 2013).

## 2. Case Series

**Figure 1:**
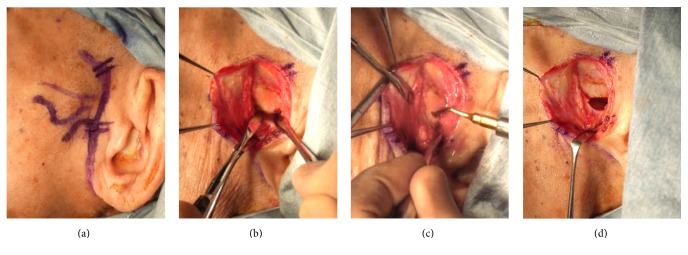
Eminectomy of the left temporomandibular joint. (a) Skin incision line. (b) Inside the TMJ capsule. (c) Incision of the eminence. (d) Following bone incision.

A total of five patients (three males and two females) were examined and found to have recurrent luxation of the TMJ (at least twice a week for 3 months). The age of patients ranged from 18 to 93 years (mean age: 72.0 ± 30.7 years). The medical histories included asthma, myocardial infarction, and hypertension. All five cases had undergone autologous blood injection (ABI) prior to surgery. However, the recurrent dislocation had occurred in all cases. Bilateral eminectomy of the TMJ was performed for two patients, left unilateral eminectomy was performed for two patients, and right unilateral eminectomy was performed for one patient (Figure 1). Two patients were examined under intravenous propofol sedation and local anesthesia. Under sedation, movement of the TMJ was confirmed. Three patients were examined under general anesthesia. Postoperatively, in one case, the patient experienced mild left ptosis but recovered a month later. One patient died from ileus one month after surgery, but any relationship between use of propofol and ileus was not confirmed. The follow-up period excluding the case that died from ileus ranged from 12 to 33 months (mean period: 21.3 ± 9.0 months). No recurrent dislocation of the TMJ has been identified at long-term follow-up (Table 1).

## 3. Discussion

Since eminectomy was first described by Myrhaug [2], a variety of treatments for the habitual luxation of the TMJ have been developed, such as ABI [6–8], miniplate eminoplasty [9], Dautrey's procedure [10], glenotemporal osteotomy [11], and botulinum toxin injection [12]. However, to date, determining which treatment options result in the best long-term results has been difficult [13]. ABI is a nonsurgical and minimally invasive treatment, and many clinicians have tried to reveal the efficacy [6–8]. Candirli et al. [8] described the benefits of ABI as being limited to patients whose joints dislocated very frequently (at least twice a day). According to Undt et al. [14], eminectomy should be considered in patients of advanced age, in patients with neurologic disorders that increase muscular tension, or in those suffering from epilepsy.

Eminectomy is usually performed under general anesthesia [2, 3]. However, rarely, reports of eminectomy under local anesthesia and sedation have been described [3–5]. From our case series, three cases were performed under general anesthesia and in two, who were high risk for general anesthesia, intravenous propofol sedation and local anesthesia were used. No anesthetic complications were observed in our case series. As many patients with chronic TMJ dislocation are elderly and have other health issues, performing eminectomy under local anesthesia plays a very important role for these patients [15]. Kitamura et al. [4] reported the advantages of eminectomy compared to other open surgeries in terms of recurrence rates. They followed up 16 patients after surgery for 6 to 36 months and none had recurrence. Recurrence was also not seen in our case series although one patient died soon after the procedure due to ileus. Lastly, the use of sedation allows patients to obey operator commands [5] so that volitional joint movements can be observed during the procedures.

Based on our experience and two other small series in the literature, eminectomy with sedation and local anesthesia can be considered for some patients and might be a good option in elderly patients in whom other health conditions could prohibit general anesthesia.

## Figures and Tables

**Table 1:** 

Case	Age	Sex	Systemic disease	Anesthesia	Side	Follow-up (months)
1	81	F	Dementia	Local	L	33
2	88	F	Pneumonia	Local	L	12
3	93	M	Hypertension	General	bilat	23
4	18	M	None	General	R	17
5	80	M	Heart disease, asthma	General	bilat	—
